# Porcelain as a Filling Material, Its Qualities and Manipulation

**Published:** 1903-08-15

**Authors:** Henry C. Raymond


### DENTAL REGISTER.

**Vol. LVII. August 15, 1903. No. 8.**

### PORCELAIN AS A FILLING MATERIAL, ITS QUALITIES AND MANIPULATION.

BY HENRY C. RAYMOND, D.D.S.

Read before the Michigan Dental Association, July 8, 1903.

During the last five years we have read and heard much of porcelain as a filling material. In the last two years especially, our journals have contained many articles on the subject, and there has hardly been a meeting of importance at which papers and clinics have not been given. In fact, I may say that the most *potent* word in the dental profession to-day is "Porcelain." It seems to occupy the first place. Every thing pertaining to it is eagerly sought and read, and it forms the basis for the most interesting and lively discussions at all our dental gatherings. Seeing that so much has been said and written about it, it is not so easy a matter to write a paper that will both interest and instruct, as it would be if the field had not been so largely covered.

But though it has been so prominently before us, I believe that a large majority of the profession yet know very little about it from a practical standpoint. There are many who yet doubt its practicability as a permanent filling material, some from honest motives, others because they have not the energy to enter into a new field. There are many attempting the work, but are only half succeeding, from a lack of knowledge of definite methods, and there is doubtless a great number who have faith, but are hesitating on which road to start, the high-fusing or low-fusing, the impression, method of getting the matrix, or the direct burnishing. Knowing this to be so, and believing as I do that in porcelain we have the best tooth preserver at our command, and a material that every intelligent dentist should be able to successfully avail himself of, will, I trust, enable me to say something that will convince some who are skeptical, encourage those who need it, and help to start in by the simplest and most practicable methods those who are anxious to take it up.

Porcelain is no longer an experiment. The years service it has given, and the manner in which it has and is saving teeth that all other materials have proved a failure in, indicate that it is almost, if not quite, an ideal restorer of lost-tooth substance. It honestly merits the high position it holds with those who are benefiting their patients by its use. Its wide range of application, its compatibility, its preservative qualities and permanency, with its wonderful harmony with tooth structure and color, are making for porcelain the position it holds to-day—a position in which it will become more firmly entrenched as time goes on. Its range of application is almost unlimited, in fact the individual operator's ability to apply it is the only limit to its use. By the skillful operator it can be used wherever gold or any of the plastics can be used, and more, it can be successfully used in many places where gold or other materials can not be used. However strong this statement may appear, it is fully borne out by the experience of those who are skillful in its application. In the teeth of old and young it is equally to be depended upon. Especially in childhood and youth, in those frail, so-called soft teeth, that crumble away from gold or amalgam, and seem to defy all attempts at permanent salvation, porcelain may be depended upon to save them. It is the only material that will save teeth for many years by filling that would otherwise have to be crowned. The importance of this can hardly be over-estimated, for however well a tooth may be crowned, it is rarely so satisfactory an operation as is a well-filled tooth, especially when it is remembered that the pulps in most crowned teeth must be devitalized and removed, which in many instances would not be necessary if restoration with porcelain were resorted to instead of crowning. In the preservation of the teeth permanently, porcelain certainly seems to stand at the head. This can not be doubted by any who have had opportunity for observation. Those who have been doing this work a number of years, both in this country and abroad, say they do not get recurrence of decay around porcelain fillings, and do not expect it. One operator, whose integrity can not be doubted, makes the statement that during the ten years he has been using porcelain, he has not once seen recurrence of decay around the inlays, and I can fully substantiate that by my own experience extending over a number of years. In many mouths the teeth filled with porcelain have been the only ones being preserved, while other teeth in the same mouths have been filled and refilled with gold, only to result in failure. Why there is not recurrence of decay, no one has yet satisfactorily explained. There are theories in plenty, and I will not take up time in more theorizing, further than to say, that probably the compatibility of porcelain with tooth structure, with the cleanliness of its non-porous glazed surface, brings about that environment that exists in mouths when the teeth become immune from caries. This feature of compatibility I can not too strongly commend; it means so much to us. It enables us to save many pulps that would otherwise have to be destroyed if metal were used as a filling. Teeth that are persistently annoying under metal fillings and sensitive to thermal changes, even in some of the shallow cavities, are almost at once rendered comfortable when porcelain is used. So marked is its compatibility, it almost seems to act as an anodyne. In large compound cavities, where sufficient anchorage can not be obtained to insert a gold filling without encroaching on the pulp, and frequently necessitating its removal, porcelain can be used without endangering the pulp in the least. This is undoubtedly partly due to its conducting quality being almost analogous to that of the teeth, and so enabling it to meet the thermal changes much in the same manner as they do.

Much more might be said along this line, to show why porcelain should have so strong a claim on us, possessing as it does all those desirable qualities necessary to save the teeth. I will pass to the feature possessed by porcelain which first attracted me toward its use, viz: Its cosmetic or esthetic value, its harmony with tooth color and structure. Though this feature is a very attractive one, I think it not out of place to speak of it last, when we consider the more important qualities porcelain possesses. To imitate nature as closely as possible should always be our foremost thought. This we can do with porcelain as we can do with nothing else. It certainly can only be the familiarity of our patients and ourselves with the use of gold that permits its use in the anterior teeth, with its consequent unsightliness and disfigurement. Porcelain, with its beautiful possibilities in matching the teeth and rendering them pleasing to the eye, must, I am sure, appeal to all without further commenting upon it. The saving of fatigue to both patient and operator is something that should strongly commend the use of porcelain to us. It does this in a large degree, and with nervous, frail patients and hard-worked operators, you well know what an advantage it must be.

Following closely on the question, shall we use porcelain? naturally comes the much discussed question, shall it be high-fusing or low-fusing? Considering the wide difference of opinion on this point, it is not surprising that it should be a stumbling block, not only to those who are not far enough advanced in the work to judge for themselves, but also to those who have not commenced. As I am only interested in trying to place before you the manner and means by which the best results may be obtained, I do not hesitate to state that I am strongly in favor of the high-fusing porcelain, and will try and show from clinical experience why we should use it, to be the most successful from a mechanical and artistic standpoint. The principal claim made by low-fusing porcelain advocates is that it is easier to burnish a gold matrix than it is to burnish one of platinum, and that closer adaptation can be obtained with its use. It may be, and probably is, a little easier to burnish gold into a cavity than platinum, but excepting in the simplest cavities it is much more difficult to remove the gold matrix without changing its form than it is one of platinum. The statement made by one or two Eastern men that there are cavities into which gold can be burnished giving perfect adaptation in all parts of the cavity, and that platinum can not be used with the same success in such places, is untrue and entirely without foundation, for any one skilled in the use of platinum for a matrix can get just as perfect adaptation in all parts of the cavity and at the margins as can possibly be obtained with gold. In fact, the finished inlay for the most difficult cavity made from a platinum matrix will show closer adaptation than one made from a gold matrix. This was conclusively shown at the recent meeting of the Chicago Odontographic Society, when Dr. Wm. T. Reeves, of Chicago, made inlays of high-fusing porcelain for two teeth in which cavities had been specially prepared either for or by Dr. Ottolengui, of New York, who is probably one of the strongest advocates of the gold matrix and low-fusing porcelain. The result showed that not only could good adaptation be gotten from the use of platinum, but that much better adaptation could be secured than by the use of gold, for the finished inlays made by Dr. Reeves of high-fusing porcelain were more perfect in every way than those made of the low-fusing porcelain. No doubt one reason for this is, that owing to the greater stiffness of the platinum the matrix can be removed without change in form, which is a most difficult matter to accomplish with the gold. I think the claim in favor of gold to give closer adaptation may be dismissed, for experience has proved to the contrary. But supposing that just as perfect adaptation could be secured with the gold matrix, there are disadvantages connected with its use that do not apply to platinum, which does not require the use of investment material. There is just enough stiffness to the metal to admit of free handling without changing form, while the the porcelain body is being built in. It is also much easier to return the work to the cavity for a guide in contour, or for a reburnishing as is sometimes necessary. The handling of a platinum matrix simplifies the work considerably over the use of a gold one. In speaking of the porcelain itself, I will be as brief as possible, for time will not permit me to go as fully into it as I would like. The subject is a most interesting one, and could well form the basis of a paper in itself.

The advantages possessed by the high-fusing porcelain are enough in themselves for us to decide in its favor, even if the method of using it were more complicated than that of the low-fusing. The question of strength it is not necessary to go into, as both the high and low-fusing porcelains of the best grade are strong enough for all our requirements. The difference in shrinkage between the two is considerable, there being very much less in the high-fusing, which is a decided advantage, as fewer bakings are required, and it is easier to gauge the work. This is, of course, most noticeable in large contour fillings. The two most important points in favor of high-fusing porcelain are its greater translucency and more life-like appearance in color and texture, though an advantage almost as important is that it will retain its form under heat, and does not run and flow out of shape as the low-fusing material does. One does not run nearly so much risk in burning out the color in the high-fusing. With the low-fusing very little heat above the actual fusing point will entirely destroy the color as well as the form. High-fusing porcelain does not change color in the mouth as the low-fusing enamels are apt to.

When the above-mentioned advantages possessed by the high-fusing material are taken into consideration, and when it is considered that the low-fusing porcelain has no advantage over it, it is, I think, evidence enough why the former should be chosen by those who desire to obtain the most satisfactory and harmonious results in their work.

There is no doubt that beautiful work can be done with the low-fusing enamel by those well experienced in its use, and for Dr. Jenkins, its leading exponent, I have the greatest regard and respect, for I think it is largely through his effort that the great interest and revival in porcelain inlay work has been brought about. But though I admit this, I am satisfied that still better work can be done with the high-fusing porcelain, and believe that the technique of its manipulation is simpler and the results more definite. I have seen the work of experts in both materials, and have worked them both myself, but I have seen few inlays made of the low-fusing porcelain in which the adaptation and contour was so good as it was in those made of the high-fusing material. The difference between the two in translucency and life-like appearance is very marked indeed.

Having decided on using a high-fusing porcelain, we should, before selecting our material, have some knowledge of the principal qualities it should possess to give us the best results. When high-fusing porcelain is spoken of, it is understood to mean one that fuses above the melting point of gold, but it does not necessarily mean that those bodies fusing at the highest degree of heat are the best for inlay work; though it may seem that the higher the fusing point the greater the translucency. Extensive experiments have shown that for inlay work the best results are obtained with an enamel body that fuses somewhere between 2100 degrees and 2400 degrees Fahrenheit. You may have a high-fusing body that falls far short of what is required, and some I have used are wanting in the essential points, both in the working of the material and in the finished inlay. What we need is a body that is finely ground, so that it will pack closely, and have the minimum amount of shrinkage, a body that can be easily shaped and carved, that will stand up and keep its form and color under heat, and will not disintegrate or fill with air bubbles with repeated bakings. Further, there should exist strength and density compatible with translucency, and the final baking should show a beautifully-glazed enamel surface, entirely free from any granulated appearance or pits.

Brewster's enamel bodies come nearer filling these requirements than any I have used. I do not hesitate to mention the name of this manufacturer, for he has probably made more extensive experiments in this line than any one, and that, too, in collaboration with practitioners who were seeking the best. He has by the excellence of his product created a demand, when little existed, by giving the profession at a time when it was most needed, an enamel body that has greatly simplified the work in this field. In the making of porcelain fillings, two grades of fusing material should be used, a foundation body and an enamel body. The foundation body should fuse at a degree of heat at least 200 degrees higher than the enamel body, and is used as its name indicates for the foundation or first baking, to insure against change in form during subsequent bakings, which would take place were only one grade of fusing material used. This would be very marked in large compound fillings where a large mass of porcelain all fusing together would, by contraction, draw the margins of the matrix in and spoil the adaptation. As I am going to give a clinic in this work, and I can then show cavity formation and the several steps in inlay construction, as well as the method of getting colors, better than I can give by description alone, I will not take up your time by going minutely into details here. My method of forming the matrix which I have described in other papers is the same as taught by Dr. Reeves, whose system of getting colors he gave in a paper which he read at our last State meeting.

I will therefore give only the underlying principles to be followed from the formation of the cavity to the setting of the finished inlay. In shaping the cavity, cutting must be proceeded with until sound enamel and dentine is reached, always bearing in mind that there must be no undercuts. If in removal of decay undercuts have been formed, they must be filled up with cement. Any device of self-locking form or retaining walls are totally unnecessary, in fact, they must be strictly guarded against, or it will be impossible to withdraw the matrix without changing its form. A decided base or seat must be made however, to keep the inlay from rolling around and slipping out of place when being set. Shallow cavities should be cut deep enough to prevent the cement from reflecting through the porcelain. The margins must not be beveled from the outer surface, but cut to a knife edge, made perfectly even and smooth, and polished wherever possible. There is no more important step in the work than that of having perfect margins, and it should always be kept well in mind.

In approximal cavities in anterior teeth extending to the incisal edge, it is well to cut the lingual wall in excess of the labial to aid the filling in resisting the outward force exerted during mastication. A strong feature in connection with porcelain work is that in approximal spaces we must get sufficient separation before starting. This is very essential. This gives opportunity for perfect contact and contour, and permits the gingival margins of the gum tissue to retain their normal aspect, all of which is of vital importance for inducing cleanliness, and for the comfort of the patient. I might say that sufficient space means that we must at least get as much as would be necessary to make a perfect gold filling, in some cases it means a little more. Don't be afraid of getting too much space in which to work. In approximal cavities, get it by wedging; in labial and buccal cavities extending up to and beneath the gum, get it by pushing the gum away with gutta percha. If in preparing a cavity it is considered necessary to apply the rubber dam, before doing so get the color of the teeth, for after the dam has been over the teeth a short time they dry out and become several shades lighter, which is very misleading, and results in an imperfect match in the inlay.

For the matrix I use platinum, one one-thousandth of an inch in thickness, and in nearly all cases burnish directly into the cavity. Some operators advocate taking an impression of the cavity and swaging the matrix on casts or dies by the water-bag method or rubber cushion. Sometimes fairly good inlays are produced by this method, but rarely is the adaptation so good as could be gotten by direct burnishing. I have discarded all such methods for general work and can see no advantage in them. They are rather a disadvantage, for it is a complicated and roundabout way of doing something that can be much better accomplished by the simpler, quicker and more accurate method of direct burnishing. Those who have tried both methods will, I think, see at once the force of this argument.

The main points to be observed in burnishing are perfect contact in all parts of the cavity, to have smooth and accurate margins, and to be sure in removing matrix from the cavity not to spring or pull it out of shape. How to accomplish this I can show better by demonstration. Always use a piece of platinum as large as is practicable. as this will facilitate handling. By freely extending it over the margins you will produce the form of the tooth, which in contour work is of great assistance in building out the porcelain. In compound cavities only is it necessary to return after the first baking to be reburnished. When much lost tooth structure has to be restored, I "try in" the partially-finished inlay for guidance in contour, but the only cavities in which I reburnish are those in bicuspids and molars involving the approximo-occlusal surfaces, These are the most difficult of any cavities we meet with, and it is not an easy matter here to get an inlay that fits, owing to the shrinkage of the large mass of porcelain which draws the margins of the matrix towards the center. To overcome this, after I have baked in the foundation body, of which I use as much as possible, always being careful not to allow it to extend to the margins, I return to the cavity and thoroughly reburnish to the margins again. Here comes the great advantage in using two grades of fusing material. I build up the rest of the inlay with the enamel body, which fuses at a much lower degree of heat than the foundation body. The temperature required to fuse the foundation body is not again reached, consequently there is no further shrinkage of that portion of the inlay, and it holds the matrix in the form into which it has been reburnished. I do not remember that this method of controlling these cases has been advocated by any one else, but after considerable experimenting I have found it is the only way to get satisfactory results.

With the matrix ready, comes the building in of the porcelain, and it is in this part of the work, with the matching the color of the teeth, that our ability is tested in being able to discriminate between the different shades and colors to be found in the teeth. It is here where our artistic sense must be brought into play. As I said earlier in my paper, I get my colors by building up in layers. To do this successfully, one must learn to find the underlying colors which exist in all teeth. They are in the dentine only and reflect through the enamel, which varies very little, being of a neutral shade. Though at first it may seem difficult to detect these underlying colors, you will be surprised how, after a little practice, you will be able to detect two or three different shades in teeth that at first sight seemed just one plain color. What looked like a simple yellow tooth will, when closely examined, be found to have a gray or blue tint or both mixed in with the yellow, and to produce the natural translucent effect we are working for, for these colors must appear in the inlay. This can only be accomplished by building up and baking in layers the different colors called for, and blending as required, remembering that the different shades of any one color are produced by the depth of the layers used. The thicker the layer the deeper the shade, and the reverse. For the anterior teeth in approximal cavities inlays can be made in this way, having the yellow color which generally appears at the neck of the teeth, and gradually shading down to the delicate gray or blue tint at the incisal edge, giving most natural and beautiful results, and which cannot be produced in any other way. It is entirely unnecessary to mix two or more colors together to get a given shade, and is undesirable, as the effects would be opaque and china-like. With practice any effect we desire may be produced by the building up in layers which not only gives the most harmonious results, but also gives that much desired translucency, and in a large measure does away with the shadow problem by breaking up the rays of light.

I can hardly leave this question of shades and colors without speaking of the use of primary colors, which are really high-fusing bodies laid on like oil paints. When we desire to carry out in the inlay a stain which is in the natural tooth, or when deeper colors or shades are required than can be got with the porcelain bodies, these paints are invaluable, as nature can be so closely imitated with them that it is almost impossible to detect their use.

After the last baking, the platinum is stripped off and the reverse side of the inlay etched with hydrofluoric acid. It is now ready for the final stage of the work, the setting, and it is just here where failure will come if we are not careful and thorough. All the good work and care that has been expended up to this point may go for nothing from faulty manipulation in the setting. I am frequently asked how I get my inlays to stay in. To that I can only reply that I observe strict rules in setting them. Experience and time have proved that inlays can be retained in any position without the aid of pins baked in the porcelain or cavity extension of any kind, if there is perfect adaptation in all parts of the cavity, and they are set with the cement under pressure, taking care of course to use a reliable cement, and keeping it dry till perfect crystallization has taken place. Whenever possible, the rubber dam should be applied and the greatest care taken in the manipulation of the cement. Pressure may be obtained by the use of the wedge, ligatures or in occlusal cavities by allowing the patient to close the teeth on the inlay with a cushion between. It takes about thirty minutes for the cement to properly set.

After the surplus cement is trimmed off, it should not be necessary to grind and polish the margins of the inlay as is advocated by some. If the cavity margins are properly prepared, and the requisite care is used in building the porcelain to the margins of the matrix, the inlay will fit flush and no finishing will be required. It is claimed that the enamel bodies may be ground and polished, which is true, but when it is done you have a far different surface to the glazed one as it comes from the furnace. When the surface is ground off it does not matter how well it may be polished, you destroy one of the strongest claims made in favor of porcelain, viz: Absolute cleanliness from its glazed, non-porous surface.

To satisfy yourself on this point, examine with a magnifying glass a piece of porcelain that has been ground and polished. The occlusal surface may be ground if necessary, as it will be kept clean by friction, but the grinding of all other surfaces should be avoided as much as possible. As the cement will darken the inlay a trifle, it should be a shade lighter than the tooth, though it is well to always use a cement as near the color of the tooth as we can get.

Those who are doing this work, but have not tried the method of getting colors by building up in layers, may think it a very difficult process. They will find, however, if they thoroughly understand the principle, and faithfully work it out, that the satisfactory results obtained will more than repay them for the extra time it will at first require.

To those who are not at all familiar with this work, this paper may give the impression that filling teeth with porcelain is a long and complicated process. Such really is not the case, though, to be a successful worker in this field, one must have delicacy of manipulation, skill and patience. We do not all possess these qualifications in an equal degree, but there is not one here who could not acquire them in at least a large measure.

Before attempting to work even on the simplest cavities in the anterior teeth, I strongly advise practicing on teeth out of the mouth. Of course, there will be failures in the beginning, in fact I know of no work that is so exacting in demanding the closest attention to the simplest details. Patience you must have. But patience and preserverance will win out, and you will be surprised how in time you will be getting pleasing results, and will soon find yourself succeeding in filling with porcelain cavities in teeth that at the beginning seemed utterly impossible.

Porcelain work is clean and beautiful, and should give us more moral tone, for we are getting nearer to nature by using it. We must observe certain laws or rules in its use which will make us more careful, and raise the standard of our work generally. Then, too, I am sure that there is nothing in life that gives more genuine satisfaction than achievement, or that which is accomplished by the aid of our best efforts. Filling with gold has at times been strongly advocated by some of our most skillful operators and valued teachers, if only for the reason that its use calls for the highest skill at our command.

In no less measure does porcelain work do this. It surely demands the best that is in us, and just as surely does it repay us in the beautiful results obtained. There is no work I have ever done that has given my patients and myself more genuine satisfaction than porcelain inlays.

### DISCUSSION.

Dr. W. H. Cudworth, of Milwaukee: The essayist has so skillfully handled the subject that he has left little to be said in the way of amplifying the subject. I can't altogether agree with his conclusions as to the relative merits of the high *versus* the low-fusing bodies. I have no doubt the essayist is sincere when he says that he can get much better results when using the high-fusing bodies, and can't believe that the low-fusing bodies have any claim to merit in comparison.

I have used both bodies, and have reached the conclusion that with low-fusing bodies—and by this I mean the bodies fusing not lower than the Jenkins bodies—I can get the most satisfactory results. I don't know how you are to harmonize these statements unless you conclude that we are both right, and that this question is largely one of personality. I am in accord with the low-fusing porcelains and can work them successfully, while the essayist has directed his attention toward the so-called high-fusing porcelains.

I have for the past five years used the Jenkins porcelains, and for all kinds of contour fillings, including restoration of the proximo-incisal angles, the severest tests to which any porcelains may be subjected. The secret of their use lies in the proper attention to the "firing." These porcelains may be blended in the same manner as the higher-fusing porcelains. They can not be subjected to the same heats without complete fusing and a tendency to spheroiding. I confidently believe that the Jenkins porcelains are stronger when properly fused than the Brewster.

In regard to the production of the matrix, I would say that the form of cavity preparation has much to do with the material used as a matrix. I believe I can produce a more perfect matrix with gold foil from the cavities I cut than I could do with platinum foil. I believe a gold matrix can be removed from the cavity easier than a platinum matrix, and with less change of form in so doing. My method is to use a sufficiently large piece of gold foil to take the cavity and a large portion of the tooth adjacent to the cavity. After it has been pressed and burnished to place, fill it with a hard wax, and then it can not change form in removing from the cavity. After removing from the cavity I insert in a mixture of equal parts of plaster and silex, being careful to get an accurate investment of the cavity margins.

In fusing the first porcelain body I am careful to not completely fuse it to a gloss surface; if this is done there will be spheroiding and a drawing away from the lateral walls. The proper fusing of porcelain is difficult but essential to success.

In cutting the cavities it is important that good, clean and definite margins should always be made. It is not necessary that the cavities should be saucer-shaped. There should, of course, be no decided undercuts, but the side walls may be more nearly parallel than when the platinum matrix is used. The gold will fold on itself or stretch so as to take in considerable angles.

As to the recurrence of caries about porcelain inlays, it is a fact that it does not seem to recur as with other filling materials. It may be due to the fact that the cement used in setting the inlay seals the dental tubules and thus prevents the solvent action of the pulp exudates. The non-conducting property of porcelain permits free use of the teeth without pain, and thus the teeth are kept cleaner by mastication. Less than two percent of the inlays I have set in four years have failed, and I have set over three hundred inlays in that time. There is no insurmountable difficulty in working porcelain; it is painstaking work and requires patience and skill. Just in proportion as these attributes are possessed, so will be the results when judgment is used in the selection of cases. No one should attempt to take up this work without some preliminary practice or technical work on teeth out of the mouth. It, however, requires no more time to learn than any other method of filling teeth. It needs practice to make one capable.

I don't think there is any advantage to be gained by baking pins into the inlays, as they weaken the structure of the inlay. I do, however, think some form of mechanical retention is good. I always prepare my cavity so that it has a definite form so as to facilitate setting. An approximately round or oval inlay is very difficult to set. I prefer the Harvard Cement, as it can be mixed thin enough to set slowly and give time for satisfactorily adjusting the inlay before it begins to set. The dam should be used if practicable to secure dryness and kept in place until the cement has set completely. This will sometimes require a half hour. After it has set trim the cement down to the tooth and inlay surface. In time a little of the cement will dissolve away, but the joint line will not show if a perfect adaptation has been made.

The secret of light refraction and color lies in the manner of using the porcelains rather than in the porcelains themselves. The Jenkins bodies should be put together in layers, as Dr. Reeves suggests, rather than by mixing before insertion. The basis of most teeth is yellow, and this is the color to use first, then lay in the superficial colors as seen in the natural teeth you are to match. We must necessarily study the porcelains we use to get the best results. I believe no other filling material is better so far as usefulness and artistic results are concerned.

Dr. J. M. Thompson, of Detroit: I have had considerable experience in using porcelain as a filling material, and from that experience I would say that the dentist who assumes to do this work without some preliminary experimental work has a great deal of assurance, and will probably either drive away his patients or soon conclude that porcelain has no useful place as a filling material, and give it up before he learns how to properly manipulate it.

In my judgment, the discovery of porcelain inlays as fillings is of equal importance with that of cohesive gold.

So far as my experience goes, I would say that I have never seen a low-fusing inlay that could compare favorably with one made of the high-fusing porcelains.

The foundation body usually should be yellow, as has been said, to give the natural-tooth color, but it should also be in sufficient amount and of such tone in color as to prevent the cement used from discoloring the inlay.

One of the most important features of this work, next to cavity preparation in the proximal cavities, is that plenty of room should be secured not only for securing the matrix, but for inserting the inlay when completed. Many failures to secure proximal contact have resulted from neglecting this point.

There is considerable difference in the flexibility of the platinum supplied for the matrix; sometimes it works as soft as lead, and again it is stiff and inflexible. A soft platinum may be made stiff by burnishing.

In most cavities it is necessary to replace the matrix in the cavity after the first baking and reburnish to the cavity margins. I have sometimes gone so far as to make a new matrix and refit the inlay inside of this matrix to insure a more perfect fit. I generally etch the back of all inlays with hydrofluoric acid, but have used Land's low fusing cement with apparently good results. In setting the inlay always set it under a continuous pressure, which should be applied carefully so as not to fracture the inlay. If the inlay needs to be ground, don't attempt to polish it, but return it to the furnace for a fire glaze; always do this before removing the matrix.

I don't approve of the suggestion made by Dr. Cudworth of filling the inlay with hard wax, as the wax will shrink and draw the matrix away from the cavity walls. I can't approve of his cavity preparation, but cut all cavities so they will readily draw from the cavity, being careful not to make such preparation as will make a very thin marginal edge.

Dr. C. H. Warboys, of Albion: I have experimented considerably with porcelain in teeth out of the mouth and find I can get fairly good results until I come to work in the tooth in the mouth. The statements of the essayists and the gentlemen who have discussed it dishearten me, as I am unable to get such results as they picture to us. I was considerably handicapped in my early researches, because I used a very inferior grade of low-fusing porcelain and a gas furnace. My inlays have been too much on the speroidal order to be either useful or artistic. I shall take heart and try with the more perfect appliances and methods suggested here to-day. Some of my fillings have failed because they came out.

Dr. C. H. Oakman, of Toledo: I have had some success and a few failures—I shan't say what proportion—I have been able to get a more secure anchorage by grooves cut in the inlays, and also in the tooth after the matrix has been made. I sometimes secure good color results by mixing the colors on the slab before laying in the matrix. This method of securing proper colors will, I imagine, require a degree of artistic skill greater than is required by baking in the strata as indicated. A good "mixer" could do this. I have had much difficulty in overcoming the shrinkage in large masses of porcelain, such as are sometimes required in molar fillings. If a large mass of porcelain is placed in the matrix and fused it is liable to contract and pull the side of the matrix away from place.

DR. E. C. MOORE, of Detroit: I have a patient for whom I made an inlay fifteen years ago. It has fallen out three times since it was first inserted, but each time it has been promptly replaced, and the tooth has not decayed a particle since it was first filled.

MR. R. BREWSTER, of Chicago: I thank the essayist for his kind remarks, also the Association for the privilege of the floor. In regard to light refractive effects, it is necessary not only that the porcelain contain certain elements, but that more than one grade of porcelain be used.

I began manufacturing porcelains for inlays on the supposition that low-fusing porcelains were indicated for this work. I have gradually raised the fusing point at the suggestion of my dental friends who were experimenting with inlay work. I have found that there is a limit from the practicable standpoint as to the higher degrees. I am now making rods of high-fusing porcelain which may be cut in sections and placed in the center of large inlays so as to prevent the great contraction and consequent distortion of the matrix in fusing a considerable mass of porcelain.

The finely-ground bodies are stronger than those coarsely ground, because there is a more intimate coalescence of the molecules of porcelain. If, however, there is complete fusion the bodies will be correspondingly hard and strong. This may be demonstrated with the Jenkins bodies which are hardest and strongest when overfused. But, of course, this porcelain, as well as others, is likely to take on the speroidal form when overfused. The maximum strength will be found in porcelains which fuse from 2,200 degrees to 2,400 degrees, and they must be completely fused.

I am now making a low-fusing body which fuses a little higher than the Jenkins body and will not speroid when properly used. It will keep up a sharp angle even when overfused.

Would recommend the making of models from impression of the cavity, over which the soft, thin platinum can be swaged easily. Would also recommend the small modeling outfit I make for the learner to practice on, that he may get needed practice in the art.

DR. RAYMOND, closing discussion: The main object I had in writing this paper was to give the beginner such suggestions as would encourage him to go on with the work, when he had become discouraged because he had taken up the work by faulty methods. My experience and judgment also incline me to the opinion that the beginner will make better progress with the platinum matrix. Investment materials are liable to change form under heat and so spoil the fit of a matrix. Therefore, I see no use in using a gold matrix which is so thin that it must be invested to prevent fusing.

It is possible by great care to get sharp and angular contours with low-fusing porcelains, if one is very careful in "firing", but you can always get them with high-fusing porcelain without being so careful.

It is not necessarily true that we are always compelled to make saucer-shaped cavities when using the platinum matrix; we may make step cavities, as in the proximal surfaces of the molars and bicuspids. We can and should always make the cavities with a somewhat angular seat to facilitate setting and to assist in retention. We should always avoid a cavity preparation which will give a thin edge at the occlusal surface, where the stress of mastication may cause its fracture.

Some inlays loosen and come out of the teeth as well as some fillings, but a well-adapted inlay that is properly set is no more liable to be dislodged from the tooth than any other filling, and the chances are that it will stay as long and preserve the tooth better and with more comfort to the patient than any metallic filling.

In making large proximal fillings the matrix should be largely filled with high-fusing body and then replaced in the cavity, and the matrix burnished up against the walls and then finished with lower grades of enamels. This will insure a fit and prevent the possibility of the matrix changing form, as the first porcelain body will not change under the heat required to complete the firing.

I do not think it is at all necessary, and it is really objectionable, to cut undercuts in the inlay or in the tooth to assist in attaching the inlay with cement. A properly-fitted and etched inlay will not leave even a saucer-shaped cavity if proiperly cemented to place.

I somet mes take an impression of a distal cavity in the molars and start the matrix from this, but I always complete the burnishing in the cavity of the tooth.

The suggestion that the beginner start his work on the tooth out of the mouth is good, for the reason that he has much to learn as to technical details in manipulating the matrix material as well as in firing the porcelain. When it comes to matching colors for the mouth he will need to work from the mouth.

